# Reflection over compliance: Critiquing mandatory data sharing policies for qualitative research

**DOI:** 10.1177/13591053231225903

**Published:** 2024-01-28

**Authors:** Annayah MB Prosser, Ralph Bagnall, Nina Higson-Sweeney

**Affiliations:** University of Bath, UK

**Keywords:** ethics, mandatory inclusion of raw data, open data, open research, open science, qualitative methods

## Abstract

Many journals are moving towards a ‘Mandatory Inclusion of Raw Data’ (MIRD) model of data sharing, where it is expected that raw data be publicly accessible at article submission. While open data sharing is beneficial for some research topics and methodologies within health psychology, in other cases it may be ethically and epistemologically questionable. Here, we outline several questions that qualitative researchers might consider surrounding the ethics of open data sharing. Overall, we argue that universal open raw data mandates cannot adequately represent the diversity of qualitative research, and that MIRD may harm rigorous and ethical research practice within health psychology and beyond. Researchers should instead find ways to demonstrate rigour thorough engagement with questions surrounding data sharing. We propose that all researchers utilise the increasingly common ‘data availability statement’ to demonstrate reflexive engagement with issues of ethics, epistemology and participant protection when considering whether to open data.

## Introduction

Open data policies have become increasingly common in psychological journals, with the aim of improving research transparency, openness and rigour (Kidwell et al., 2016). There are many practical, legal and ethical issues surrounding opening qualitative data (for previous discussions see Branney et al., 2019; Chauvette et al., 2019; DuBois et al., 2018; Parry and Mauthner, 2004). However, considerations of qualitative research are typically absent from open data policies, even in journals which are considered ‘qualitative-friendly’ (Prosser et al., 2023a). Many open data policies operate from a primarily quantitative perspective based upon values of objectivity and reproducibility. In contrast, a hallmark of many qualitative approaches is an embrace of subjectivity and positionality (Willig, 2019), which makes reflexivity (the practice of paying attention to and reflecting on the role of the researcher within research; Berger, 2015) integral.

Positivist underpinnings of open science mandates do not easily align with qualitative approaches. For example, elements of The Journal of Health Psychology’s mandatory inclusion of raw data (MIRD) policy for empirical articles and systematic reviews raise questions for qualitative researchers. The policy suggests that raw qualitative data could be in the form of full transcripts and other raw materials, alongside additional data like codes, themes and discourse. The rationale behind this suggestion is indicated in the journal’s announcement of the MIRD, which states that ‘*all data and analytical procedures must be sufficiently well described to enable a* third *party with the appropriate expertise to replicate the data analyses*’ (Marks, 2020). Open data mandates such as MIRD are often introduced to enable ‘replication’ (Hardwicke et al., 2018), which is not usually a concern for qualitative approaches (Tuval-Mashiach, 2021). Standards of research rigour for qualitative approaches often hinge on reflexive engagement with the research question and the validity and depth of the analysis, which MIRD policies focussed primarily on ‘replication’ cannot validate (DuBois et al., 2018). Additional ethical and practical considerations are needed when considering the relevance and appropriateness of opening data for qualitative research (Karhulahti, 2023; Pownall, 2022).

This commentary aims to query the policy of MIRD in the context of qualitative research, with the purpose of provoking further discussion amongst health psychologists regarding best practice. We outline some questions that have arisen in our own approaches to opening data and consider how these concerns may be addressed in researcher and journal practices. To learn more about the authors, their open data practices, and their reflections on the current commentary, see: https://osf.io/kjgna/

## Reflecting on questions surrounding qualitative data sharing

*‘Would you like this data raw, medium or well done?’: What does ‘raw’ data mean in qualitative research?*

What constitutes ‘raw’ qualitative data is often ambiguous. Quantitative data is typically numerical and held within a ‘dataset’, whereas qualitative data sources are much more diverse, and a higher level of data cleaning is seen as standard. ‘Raw’ qualitative data (such as audio or video recordings) often contain sensitive and identifiable information such as faces, names and locations. Opening this data, without any cleaning, de-identification or anonymisation, violates ethical principles and puts participants at risk. Anonymising raw data is often necessary for qualitative projects to receive ethical approval – it is rarely possible for researchers to publish their ‘raw’ data. Journals requiring MIRD without clear clarification of the processing permitted demonstrates a lack of engagement with qualitative methodologies, which may discourage qualitative researchers from submitting their work to such outlets. Furthermore, mandates disregard the additional time and financial burden such requests may disproportionately place on qualitative researchers (Branney et al., 2019; Pownall et al., 2021). To believe that MIRD can be applied consistently across quantitative and qualitative datasets is therefore misguided.

*‘Think of the participants!’: What additional ethical considerations are needed regarding participants and data sharing?*

The principle of ‘informed consent’ rests upon a participant’s understanding of, among other issues, how their data will be owned, stored and used in open datasets (British Psychological Society, 2020). As such, the question of how best to explain the implications of ‘opening’ qualitative research data is relevant for all research participants, but particularly for groups considered vulnerable (as defined by Hurst, 2008). Vulnerable participants’ understanding of the implications of qualitative data sharing, is a key issue to consider when seeking participant consent and when mandating the publication of raw data.

After opening data, research participants’ (potentially highly sensitive) lived experiences may be reused and reconceptualised in secondary data analysis. There is evidence that some participants welcome their qualitative data being archived and reused (Kuula, 2011). However, the intersection between participant vulnerability and data sensitivity should prompt further reflection (Parry and Mauthner, 2004). Relatedly, the situated, contextual relationships between the original researcher(s) and participants are often unavailable to secondary researchers, who may in turn misinterpret the data’s original meaning (Chauvette et al., 2019; Walters, 2009).

As qualitative researchers, we should consider how we can facilitate the understanding necessary for (vulnerable) participants to provide fully informed consent for data sharing. A starting point is communicating explicitly how their data may be reused (and re-interpreted) in future research (Ruggiano and Perry, 2019). However, such communication is not necessarily straightforward (Eeckhout et al., 2023). For instance, the difficulties in communication between autistic and non-autistic people has been characterised as the ‘double empathy problem’; whereby people with different ways of seeing the world struggle to understand and therefore communicate with one another (Milton, 2012). Furthermore, asymmetrical power dynamics within researcher and research participant relationships (Haverkamp, 2005) necessarily extend to the context of open data. Participants may not be able to understand the complexity of the issues or informedly consent to their raw data being made open free from coercion. MIRD minimises the researchers’ flexibility to accommodate participant boundaries and care in research (Weller, 2023). Under MIRD, participants who (reasonably) do not consent to their raw data being opened must be excluded from participation altogether, which is a problem when considering research on minority or marginalised groups. MIRD policies may therefore reduce the diversity of research and discourage future research involving ‘vulnerable’ groups.

*‘Forever and always?’: Considering future-proofing data sharing in changing contexts*

Reflection is also needed on how changing socio-political and technological contexts may influence data interpretation and use in the future. For researchers working in areas that are politically contentious (e.g. reproductive health care, climate change, sexual minority identity) the legal and political environments surrounding our subject areas and participant groups may change quickly. Topics not controversial at the time of data generation may become quickly politicised following publication, which could have dramatic implications for the risk of opening raw data. For example, abortion in the US went from a protected right to an illegal practice after Rode v. Wade was overturned (BBC News, 2021). MIRD therefore has the potential to present risks for participants and researchers, who may become legally liable based on evidence gathered from research data. Uploading raw data today may be insufficient to fully protect participants from legal, ethical and political harms in the future.

There may also be new *opportunities* for data (mis)use in the future that will be difficult to anticipate. Large-Language Models (LLMs) such as ChatGPT are often trained on ‘openly-accessible’ qualitative data (Weidinger et al., 2021). Participants do not often consent to the use of their data for this explicit purpose, and when raw data is uploaded freely, participants have no recourse to protest this misuse. Furthermore, new technologies are making participant identification possible even following anonymisation (Barlas and Stamatatos, 2020), requiring further caution when featuring demographic information (Morehouse et al., 2023). Applying an appropriate level of caution to these complex issues is challenging if one is required to upload ‘raw’ data in its entirety.

## Looking to the future: Discussion and directions for future open research practice

In this commentary, we briefly highlighted a (far from exhaustive) sample of questions for researchers to reflect upon when considering whether to open their qualitative data. The issue of MIRD is nuanced – no singular solution will address all the issues we have raised. However, a general principle that qualitative data should be ‘*as open as possible, as closed as necessary*’ is likely more conducive to rigorous, and ethical qualitative research practice than MIRD policies. Here, we outline some further suggestions for how qualitative researchers can move forward and advocate on this issue, from the individual researcher to broader contexts.

As qualitative researchers, we must play active roles in initiating and maintaining discussions surrounding open research practice and policy. There are opportunities for this in many different roles of academic influence, including peer review, editorial roles and teaching. The dominance of quantitative research in open science means that qualitative researchers may not always be invited to participate in these discussions. We must make efforts as a community to *become* engaged and active in these conversations to truly affect change.

While individual researchers often have little control over specific journal or organisational data sharing policies, we can utilise existing mechanisms to demonstrate reflexive engagement with these issues. One avenue is through the data availability statement (DAS). The DAS has recently become a core component of many journals’ drive towards open data. Traditionally, this statement has been used to link the location of open data or provide a brief explanation for why data has not been opened (Jiao et al., 2022). However, given the importance of richness and context across qualitative approaches, qualitative researchers would be well placed to initiate a move towards a more nuanced utilisation of the DAS. We suggest that researchers could use the DAS (which is often not included in journal word counts) to demonstrate thorough reflexive engagement with principles of transparency and ethics involved in their decision regarding opening their data. Quantitative approaches to open data also agree that further engagement with the DAS is required, and that data opened should be: Findable, Accessible, Complete and Well-described (Towse et al., 2021). As a starting point, an example of an extended narrative DAS is available in Prosser et al. (2023b).

A further option is to consider publishing the data under a ‘licence’ most suited to the individual research context. The ability to ‘licence’ data in-built within many repositories (including the Open Science Framework) may provide a route around some of the issues that qualitative researchers encounter and make the conditions of data reuse clearer (Hardwicke et al., 2018). Alongside this, ‘data-centric rights and licencing ontologies’ must be developed, particularly when considering sensitive data (Grabus and Greenberg, 2019: 1). We argue that as best practice, participants should also be given the chance to informedly consent to this licence (and data sharing) at the point of data collection to ensure full transparency, although this may not always be possible or preferable. Details surrounding data re-use and licencing should also be clearly outlined by authors in the DAS.

When developing guidelines for data sharing, journals should clearly distinguish between expectations for quantitative and qualitative data, with flexibility to account for ethical considerations unique to each study. Any guidelines introduced or mandated should be coproduced in consultation with qualitative researchers and other affected stakeholders (e.g. research participants) to ensure it is appropriate to the needs of everyone involved. Directions for such guidelines are already emerging in the literature. For example, Branney et al. (2019) outline steps for open science in qualitative psychological research, including opening metadata (information about study data); this may be considered an appropriate alternative to MIRD. Adopting tougher screening and anonymisation processes before opening data (redacting any potentially identifying information) is another vital way to ensure participant protection when opening data. Future open data mandates should include recognition of and accommodations for managing ethical risk.

## Conclusion

There are ethical, methodological and political challenges to opening qualitative data, and we believe that MIRD policies fail to recognise and help researchers navigate these issues. We argue that, for both researchers and journals, evidencing thorough and reflexive engagement with identified issues surrounding opening data should be prioritised over following MIRD policies. Such policies may harm the ethics and rigour of research and pose a disproportionate risk to qualitative research projects and participants.
